# Factors Affecting Postoperative Rehabilitation Therapy Utilization After Arthroscopic Rotator Cuff Repair: An Epidemiological Analysis

**DOI:** 10.7759/cureus.36740

**Published:** 2023-03-27

**Authors:** Anthony Baumann, Thad Indermuhle, Deven Curtis, Jaime Perez, John Martin Leland

**Affiliations:** 1 Department of Rehabilitation Services, University Hospitals, Cleveland, USA; 2 College of Medicine, Northeast Ohio Medical University, Rootstown, USA; 3 Clinical Research Center, University Hospitals, Cleveland, USA; 4 Department of Orthopedic Surgery, University Hospitals, Cleveland, USA

**Keywords:** orthopedics surgery, arthroscopic rotator cuff repair, shoulder stiffness, rotator cuff pathology, physical therapy rehabilitation, arthroscopic shoulder surgery

## Abstract

Background and objective

Rotator cuff tear (RCT) is an orthopedic shoulder pathology commonly managed via arthroscopic rotator cuff repair (ARCR) after the failure of conservative treatment options. Physical therapy (PT) after ARCR is an important component of patient recovery. Postoperative complications, such as postoperative shoulder stiffness (POSS), are frequent among these patients and place a significant burden on patients and clinicians. The purpose of this study is to analyze temporal PT utilization among patients after ARCR and its potential to improve patient outcomes and examine possible factors affecting postoperative complication rates.

Methods

An epidemiological study was performed by using a large de-identified national health research network (TriNetX) within the United States to search for patients with a diagnosis of partial or complete RCT and subsequent ARCR. Data were collected on patient demographics, number of postoperative PT visits, and PT visits distribution in the early postoperative period. Statistical analysis was performed to analyze factors that impacted the utilization of postoperative PT after ARCR.

Results

A total of 21,540 patients underwent ARCR with 11,312 receiving ARCR for partial RCT and 10,228 for complete RCT. Of all ARCR patients, 6,923 (32.1%) received postoperative PT within one year of ARCR. Patients with partial RCT had a greater number of PT visits (mean ±SD: 3.85 ± 8.33; min-max: 0-110; *t *= 15.2) compared to patients with complete RCT (2.90 ± 7.97; min-max: 0-125) after ARCR (p<0.001). Patients with ARCR for partial RCT also had more visits within the first 12 weeks after ARCR as compared to patients with ARCR for complete RCT (p<0.001). Female patients had more visits than male patients after ARCR, regardless of the RCT extent (p<0.001).

Conclusion

Partial RCT and female sex are associated with increased postoperative PT usage after ARCR. Postoperative PT utilization has high variability after ARCR, regardless of the RCT extent. More research is needed to further explore the impact of PT utilization on postoperative complications after ARCR.

## Introduction

Rotator cuff tear (RCT) is one of the most common shoulder disorders encountered by orthopedic surgeons and physical therapists [1,2]. Arthroscopic rotator cuff repair (ARCR) is a common surgical intervention for RCT after other conservative interventions for RCT have been exhausted [3-5]. Among all types of RCT repairs, ARCR is becoming more common in recent years and now makes up around 75% of all RCT repairs, which has an incidence of 13.6 out of 1,000 patients [3]. Postoperative complications from ARCR cause a significant burden on patients and clinicians alike [1]. A common postoperative complication after ARCR is postoperative shoulder stiffness (POSS), which can result in increased healthcare costs and unfavorable patient outcomes [6-9]. The incidence of POSS is highly variable and ranges from 2% to 28% in the literature [4,10,11]. Furthermore, POSS has been shown to be more frequent based on various covariates, such as female sex and the extent of RCT, which may have an impact on postoperative physical therapy (PT) utilization after ARCR [9,11]. Postoperative PT is common after ARCR and is widely considered a critical component for patient recovery and improved outcomes [12-15]. Furthermore, PT has been used to manage complications, such as POSS, after ARCR [1,11,14,16]. It is possible that the utilization of PT after ARCR could impact postoperative complications and patient outcomes.

However, there still exists a debate on the exact utility of PT after ARCR [17]. While utilization of PT can be limited due to healthcare costs, some authors contend that new methods of PT delivery, such as digital PT, can provide superior outcomes with fewer visits [14,17]. Questions regarding the ideal number of PT visits to maximize outcomes while lowering costs after ARCR remain unsettled. The literature has shown efforts to examine the utilization of PT after ARCR with the examination of different variables such as insurance type, surgical technique, and patient sex [18,19]. With regard to surgical technique, postoperative PT utilization was found to be comparable between ARCR and open/mini-open RCT repair techniques, yet little data exists on how RCT extent, such as partial and complete RCT, prior to ARCR impacts PT utilization after ARCR [19]. Furthermore, while smaller studies in the literature have examined how patient-specific covariates such as age and sex impact utilization of postoperative PT after ARCR, no larger studies have confirmed or challenged their findings [18]. Thus, a large study examining PT utilization as it relates to patient-specific covariates is warranted and may help in understanding how to optimize PT utilization and improve patient outcomes. The purpose of this study is to describe PT utilization among patients after ARCR for partial versus complete RCT and examine possible covariates affecting postoperative complication rates and overall patient outcomes.

## Materials and methods

Data source

The current study used a large de-identified national health research network with data sourced from 74 healthcare organizations (HCOs) within the United States (TriNetX, a global federated de-identified health research network exempt from IRB. Data accessed on January 4, 2023). In addition, while aware of this study, the authors' institution did not require IRB approval for this study. TriNetX continuously aggregates clinical data directly from participating HCOs and also ensures extensive data quality and accuracy assessment. TriNetX does not provide any identifiers on participating HCOs; however, a typical participating HCO includes a large academic health center with inpatient, outpatient, and specialty care services.

Patient selection

We identified all patients within the TriNetX database who had a diagnosis of RCT (ICD-10: M75.12, M75.11) and documented ARCR (CPT: 29827) based on our inclusion criteria. We further categorized patients based on the extent of RCT, including partial and complete tears. For both cohorts, we collected patient demographic and PT visit data using evaluation and treatment codes (CPT: 97001, 97005, 97006, 97010, 97014, 97016, 97022, 97032, 97035, 97110, 97112, 97113, 97116, 97124, 97140, 97150, 97161, 97162, 97163, 97164, 97530, and 97750).

Statistical analysis

For continuous data, we performed independent t-tests or one-way analysis of variance (ANOVA) due to the nature of the variables. For categorical data (presented as frequencies and percentages), we performed chi-square tests to determine significance. All tests were two-tailed with an alpha level of 0.05.

## Results

A total of 21,540 patients met our study criteria, with 11,312 experiencing a partial RCT, and 10,228 experiencing a complete RCT (Table 1). The partial RCT cohort was significantly younger and comprised more female patients when compared to the complete RCT cohort (Table 1). Among all patients in both cohorts, 6,923 (32.1%) participated in PT within one year of ARCR. We found increased participation in the partial RCT cohort as compared to the complete RCT cohort [partial: n = 4,419 (39.1%), complete: n = 2,504 (24.5%), OR (95% CI): 1.98 (1.86-2.10), p<0.001]. Patients with complete RCT had a lower mean number of PT visits (mean ± SD: 2.90 ± 7.97; min-max: 0-125) compared to patients with partial RCT (mean ± SD: 3.85 ± 8.33; min-max: 0-110; t = 15.2, p<0.001).

**Table 1:** Baseline characteristics of patients who received arthroscopic rotator cuff repair for partial or complete rotator cuff tears SD: standard deviation

Characteristic	Complete tear (n = 10,228)	Partial tear (n = 11,312)	P-value
Age, mean ± SD	57.79 ± 9.61	56.19 ± 10.35	<0.001
Sex, n (%)			
Female	4,211 (41.2%)	5,296 (46.8%)	<0.001
Male	6,011 (58.8%)	5,766 (51.0%)	<0.001
Not reported	10 (0.1%)	250 (2.2%)	<0.001
Race, n (%)			
American Indian or Alaska Native	39 (0.4%)	43 (0.4%)	0.989
Asian	89 (0.9%)	120 (1.1%)	0.154
Black or African American	1,557 (15.2%)	1,347 (11.9%)	<0.001
Native Hawaiian or Other Pacific Islander	10 (0.1%)	10 (0.1%)	0.822
White	7,732 (75.6%)	8,930 (78.9%)	<0.001
Unknown race	808 (7.9%)	868 (7.7%)	0.535
Ethnicity, n (%)			
Hispanic or Latino	447 (4.4%)	567 (5.0%)	0.026
Not Hispanic or Latino	7,280 (71.2%)	9,097 (80.4%)	<0.001
Unknown ethnicity	2,501 (24.5%)	1,648 (14.6%)	<0.001

**Figure 1:**
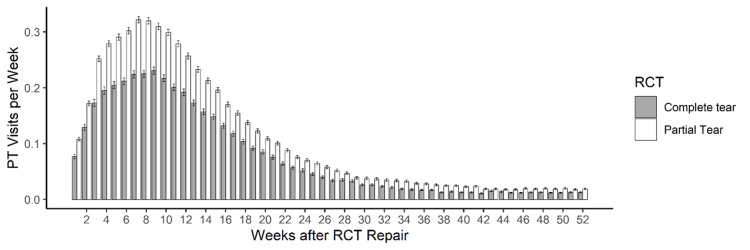
Mean physical therapy visits per week within 52 weeks of arthroscopic rotator cuff repair for both partial (white) and complete (gray) rotator cuff tears Error bars represent the standard error of the mean PT: physical therapy; RCT: rotator cuff tear

**Figure 2:**
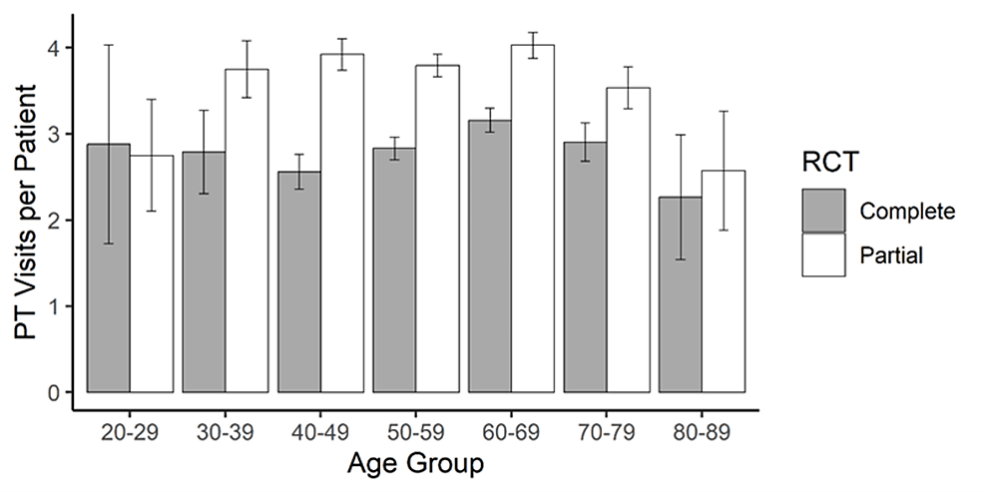
Mean physical therapy visits within 52 weeks of arthroscopic rotator cuff repair across patient age groups for both partial (white) and complete (grey) rotator cuff tears Error bars represent the standard error of the mean PT: physical therapy; RCT: rotator cuff tear

For both RCT cohorts, over 30% of visits occurred in the first six weeks and over 50% within the first 12 weeks (Figure 1). Within 12 weeks, the mean number of PT visits varied by the RCT extent, with greater mean visits in the partial RCT cohort (partial: 2.20 ± 4.51 visits, complete: 1.67 ± 4.48, p<0.001). Female patients participated in more PT visits than male patients, regardless of the RCT extent [partial: female (mean ± SD): 4.22 ± 8.62, male: 3.67 ± 8.18, t = -3.50, p<0.001; complete: female: 3.13 ± 8.37, male: 2.74 ± 7.67, t = -2.42, p<0.001]. We found no significant difference in the number of PT visits across age groups, for both partial and complete RCTs (partial: F = 1.11, p = 0.353; complete: F = 1.19, p = 0.309, Figure 2). Peak PT visits per week were seen around 8-10 weeks after ARCR (Figure 1).

## Discussion

Partial and complete RCT with subsequent ARCR is frequently encountered by orthopedic surgeons as well as physical therapists [1-3,19]. There is a great need to improve all aspects of care regarding ARCR and subsequent rehabilitation as postoperative complications remain high [20-22]. Postoperative PT remains a cornerstone of rehabilitation after ARCR and consistently demonstrates improved recovery rates and better functional outcomes [13,15]. Our study showed that the female sex was associated with increased postoperative PT usage after ARCR. This observation contrasts with the study by Brennan et al., which reported that there was no significant difference in PT utilization based on sex after four types of shoulder surgery, including RCT repair [18]. However, that study was significantly smaller than our study and did not examine partial RCT versus complete RCT [18]. Interestingly, the study by Brennan et al. did report that outcomes after shoulder surgery varied based on sex despite no change in PT utilization [18]. For example, women who underwent RCT repair had a greater preoperative disability, greater postoperative improvement in function, and greater postoperative improvement in pain as compared to their male counterparts [18]. Therefore, it remains to be seen if these outcomes based on patient sex are repeated in studies with larger sample sizes as our study did not examine patient outcomes for pain or function. More research is needed to determine the impact of patient sex and PT utilization on outcomes after ARCR.

In our study, patients with ARCR for partial RCT had a higher number of PT visits as compared to patients who underwent ARCR for complete RCT. No other studies in the literature have examined the impact of the RCT extent on postoperative PT usage after ARCR, and hence the finding that PT utilization is higher in partial versus complete RCTs is a novel finding with clinical implications. POSS, another common complication after ARCR, has also been shown to have a higher incidence in female patients and those with partial tears, which aligns with the findings of our study [9,11]. The correlation between increased incidence of POSS after ARCR and increased postoperative PT needs to be further explored.

Overall, our study demonstrated that there is a large variation in PT utilization and indicates that the actual average number of post-ARCR PT visits may be lower than previously thought. One small study, for instance, surveyed 80 patients who underwent orthopedic shoulder surgery and reported an average of 16.3 ± 13.8 PT visits after surgery [14]. On the other hand, our data showed that the average number of PT visits after ARCR for complete and partial RCT was 3.85 ± 8.33 and 2.9 ± 7.97, respectively, which is relatively lower when compared with some of the findings in the literature [14,18,19]. This low number could be due to several factors: one possibility is that a limitation inherent in the use of large, de-identified databases like TriNetX has affected the average. The limitation pertains to the fact that patients with zero physical therapy visits after ARCR could not be excluded from the analysis, whereas most other studies examining PT usage after ARCR generally only include patients with at least one PT visit. This resulted in more “zero-visit” data points in our study, thereby lowering the average. Another possible limitation involves the selection of the HCOs within the network: since TriNetX only reports on PT sessions completed in clinics within its own network, any PT visits outside of the TriNetX network would also be reported as “zero visits” in the data even if they did in fact utilize outside PT. This would further underestimate the actual PT utilization across all clinics, which could have lowered our reported average. Also, our patient population was very broad because we relied on the TriNetX database, possibly affecting PT utilization in comparison to the smaller studies. Furthermore, the range of post-ARCR PT visits in our study was unusually large for partial RCT (0-110 visits) and complete RCT (0-125 visits), reflecting that the data included outliers on both extremes. These limitations may have had the cumulative effect of lowering the averages, but should not have an impact on the data trends themselves or the covariates analyzed here; thus, while the averages should be considered with the limitations in mind, the relationships and implications of the study, such as partial versus complete tear findings, can be viewed as high-powered, data-supported conclusions.

Apart from the number of visits, our study demonstrated that the percentage of patients who received any PT at all was only 32.1%. While this finding should be considered in light of the aforementioned limitations, it is interesting given the impact PT utilization has on increasing costs of healthcare, decreased coverage by insurance companies, high copays, and limitations on PT locations [14]. The interest in the literature surrounding lowering the amount of PT visits and/or changing PT delivery methods after ARCR renders our findings part of the conversation [17]. One study has indicated that digital PT sessions, for example, were able to provide superior outcomes to conventional home-based PT after ARCR with fewer visits [17]. Considering the low average percentage and a large range of visits in the current study - the upper extreme being 125 visits in the complete RCT cohort - questions arise regarding the effectiveness and the medical necessity of excessive numbers of visits after ARCR and prompt the examination of resource utilization and distribution.

Other factors such as patient age and timeline can also be considered from the present findings. Age, for example, was shown to be noncontributory to PT utilization as there was no statistically significant difference in the number of PT visits across age groups. Timeline analysis found that over 50% of PT visits occur within the first 12 weeks post-repair. This warrants further research to examine how early vs. later postoperative PT usage can impact complications such as POSS. Strengths of our study include the large sample size (n = 21,540) and the differentiation between ARCR for partial and complete RCT, as compared to previous studies [11,14,18]. Another strength of our study is the use of the TriNetX database, which is sourced directly from HCOs as opposed to third-party vendors, like some other database studies [23]. Limitations of this study include the fact that less than half of patients after ARCR in our study received documented PT, which may skew data on PT utilization. Other limitations include the retrospective nature of this study, the reliance on documentation in the TriNetX database, and the inability to control for confounding variables. More research is needed to further examine the ideal utilization of PT, factors associated with PT utilization, the correlation between PT usage and postoperative complications, and the impact of PT on patient outcomes after ARCR.

## Conclusions

Based on our findings, female sex and partial RCT are associated with increased postoperative PT utilization after ARCR. Furthermore, PT utilization after ARCR is highly variable and not dependent on patient age. Half of PT visits occur within the first 12 weeks after ARCR. These findings highlight the need for healthcare providers to consider these factors when encountering patients who may need additional support or resources to optimize their recovery. Further research is needed to determine the impact that PT utilization has on patient outcomes and postoperative complications, as well as to explore ways to improve PT utilization after ARCR. Additionally, it is important to note that our study has some limitations, including potential confounding factors and sources of bias. Future research should aim to address these limitations to improve the validity and generalizability of the findings.

## References

[1] Longo UG, Carnevale A, Piergentili I, Berton A, Candela V, Schena E, Denaro V (2021). Retear rates after rotator cuff surgery: a systematic review and meta-analysis. BMC Musculoskelet Disord.

[2] Tashjian RZ (2012). Epidemiology, natural history, and indications for treatment of rotator cuff tears. Clin Sports Med.

[3] Zhang AL, Montgomery SR, Ngo SS, Hame SL, Wang JC, Gamradt SC (2013). Analysis of rotator cuff repair trends in a large private insurance population. Arthroscopy.

[4] Franceschi F, Papalia R, Palumbo A, Vasta S, Maffulli N, Denaro V (2011). Management of postoperative shoulder stiffness. Sports Med Arthrosc Rev.

[5] Shin JJ, Popchak AJ, Musahl V, Irrgang JJ, Lin A (2018). Complications after arthroscopic shoulder surgery: a review of the American Board of Orthopaedic Surgery database. J Am Acad Orthop Surg Glob Res Rev.

[6] Stojanov T, Modler L, Müller AM (2022). Prognostic factors for the occurrence of post-operative shoulder stiffness after arthroscopic rotator cuff repair: a systematic review. BMC Musculoskelet Disord.

[7] Huberty DP, Schoolfield JD, Brady PC, Vadala AP, Arrigoni P, Burkhart SS (2009). Incidence and treatment of postoperative stiffness following arthroscopic rotator cuff repair. Arthroscopy.

[8] Barnes RH, Paterno AV, Lin FC, Zhang J, Berkoff D, Creighton RA (2022). Glenohumeral hydrodistension for postoperative stiffness after arthroscopic primary rotator cuff repair. Orthop J Sports Med.

[9] Audigé L, Aghlmandi S, Grobet C (2021). Prediction of shoulder stiffness after arthroscopic rotator cuff repair. Am J Sports Med.

[10] Cho CH, Bae KC, Kim DH (2022). Incidence and risk factors for early postoperative stiffness after arthroscopic rotator cuff repair in patients without preoperative stiffness. Sci Rep.

[11] Baumann A, Oleson C, Curtis D, Indermuhle T, Leland JM (2023). Incidence of postoperative shoulder stiffness after arthroscopic rotator cuff repair. Arch Phys Med Rehabil.

[12] Coda RG, Cheema SG, Hermanns CA (2020). A review of online rehabilitation protocols designated for rotator cuff repairs. Arthrosc Sports Med Rehabil.

[13] Thigpen CA, Shaffer MA, Gaunt BW, Leggin BG, Williams GR, Wilcox RB 3rd (2016). The American Society of Shoulder and Elbow Therapists' consensus statement on rehabilitation following arthroscopic rotator cuff repair. J Shoulder Elbow Surg.

[14] Sabesan VJ, Dawoud M, Stephens BJ, Busheme CE, Lavin AC (2022). Patients' perception of physical therapy after shoulder surgery. JSES Int.

[15] Marinko LN, Chacko JM, Dalton D, Chacko CC (2011). The effectiveness of therapeutic exercise for painful shoulder conditions: a meta-analysis. J Shoulder Elbow Surg.

[16] Vezeridis PS, Goel DP, Shah AA, Sung SY, Warner JJ (2010). Postarthroscopic arthrofibrosis of the shoulder. Sports Med Arthrosc Rev.

[17] Correia FD, Molinos M, Luís S (2022). Digitally assisted versus conventional home-based rehabilitation after arthroscopic rotator cuff repair: a randomized controlled trial. Am J Phys Med Rehabil.

[18] Brennan GP, Parent EC, Cleland JA (2010). Description of clinical outcomes and postoperative utilization of physical therapy services within 4 categories of shoulder surgery. J Orthop Sports Phys Ther.

[19] Arshi A, Kabir N, Cohen JR, Lord EL, Wang JC, McAllister DR, Petrigliano FA (2015). Utilization and costs of postoperative physical therapy after rotator cuff repair: a comparison of privately insured and medicare patients. Arthroscopy.

[20] Le BT, Wu XL, Lam PH, Murrell GA (2014). Factors predicting rotator cuff retears: an analysis of 1000 consecutive rotator cuff repairs. Am J Sports Med.

[21] Zhao J, Luo M, Pan J (2021). Risk factors affecting rotator cuff retear after arthroscopic repair: a meta-analysis and systematic review. J Shoulder Elbow Surg.

[22] Khazzam M, Sager B, Box HN, Wallace SB (2020). The effect of age on risk of retear after rotator cuff repair: a systematic review and meta-analysis. JSES Int.

[23] Burroughs PJ, Kahan JB, Moore HG, Grauer JN, Gardner EC (2021). Temporal utilization of physical therapy visits after anterior cruciate ligament reconstruction. Orthop J Sports Med.

